# European Adrenal Insufficiency Registry (EU-AIR): a comparative observational study of glucocorticoid replacement therapy

**DOI:** 10.1186/1472-6823-14-40

**Published:** 2014-05-09

**Authors:** Bertil Ekman, David Fitts, Claudio Marelli, Robert D Murray, Marcus Quinkler, Pierre MJ Zelissen

**Affiliations:** 1 Section of Endocrinology, Department of Medical and Health Sciences, Linköping University, Linköping, Sweden; 2 ViroPharma Incorporated, Exton, Pennsylvania, USA; 3 ViroPharma SPRL, Maidenhead, UK; 4 Department of Endocrinology, Leeds Teaching Hospitals NHS Trust, St James’s University Hospital, Leeds, UK; 5 Clinical Endocrinology, Charité Campus Mitte, Charité University of Medicine, Berlin, Germany; 6 Department of Internal Medicine and Endocrinology, University Medical Center Utrecht, Utrecht, Netherlands

## Abstract

**Background:**

Increased morbidity and mortality associated with conventional glucocorticoid replacement therapy for primary adrenal insufficiency (primary AI; estimated prevalence 93–140/million), secondary AI (estimated prevalence, 150–280/million, respectively) or congenital adrenal hyperplasia (estimated prevalence, approximately 65/million) may be due to the inability of typical glucocorticoid treatment regimens to reproduce the normal circadian profile of plasma cortisol. A once-daily modified-release formulation of hydrocortisone has been developed to provide a plasma cortisol profile that better mimics the daytime endogenous profile of cortisol. Here, we describe the protocol for the European Adrenal Insufficiency Registry (EU-AIR), an observational study to assess the long-term safety of modified-release hydrocortisone compared with conventional glucocorticoid replacement therapies in routine clinical practice (ClinicalTrials.gov identifier: NCT01661387).

**Methods:**

Patients enrolled in EU-AIR have primary or secondary AI and are receiving either modified-release or conventional glucocorticoid replacement therapy. The primary endpoints of EU-AIR are the incidence of intercurrent illness, adrenal crisis and serious adverse events (SAEs), as well as the duration of SAEs and dose changes related to SAEs. Data relating to morbidity, mortality, adverse drug reactions, dosing and concomitant therapies will be collected. Patient diaries will record illness-related dose changes between visits. All decisions concerning medical care are made by the registry physician and patient. Enrolment is targeted at achieving 3600 patient-years of treatment (1800 patient-years per group) for the primary analysis, which is focused on determining the non-inferiority of once-daily modified-release replacement therapy compared with conventional glucocorticoid therapy.

**Results:**

Recruitment began in August 2012 and, as of March 2014, 801 patients have been enrolled. Fifteen centres are participating in Germany, the UK and Sweden, with recruitment soon to be initiated in the Netherlands.

**Conclusions:**

EU-AIR will provide a unique opportunity not only to collect long-term safety data on a modified-release preparation of glucocorticoid but also to evaluate baseline data on conventional glucocorticoid replacement. Such data should help to improve the treatment of AI.

## Background

Adrenal insufficiency (AI) is a life-threatening disease, characterized by signs and symptoms including weakness, fatigue, anorexia, weight loss, nausea and vomiting [1]. Estimates for the prevalence of primary AI (Addison’s disease) in Western Europe range from 93 to 140 per million inhabitants, whereas the prevalence of secondary AI (pituitary insufficiency) has been reported as 150–280 per million inhabitants [2]. Congenital adrenal hyperplasia is due, in 95% of patients, to a defect in 21 hydroxylase in the adrenal cortex and has a prevalence of approximately 65 per million inhabitants [3].

The aim of treatment of AI is to restore normal physiological levels of cortisol through administration of synthetic glucocorticoid. Conventional ‘immediate-release’ oral hydrocortisone replacement therapy has been the mainstay of treatment for AI for more than 50 years [4], typically given orally either two or three times daily. Although this conventional replacement therapy extends the life-span of patients with AI compared with untreated patients, it results in non-physiological spikes and troughs of cortisol over a 24-hour period [5,6], and life-expectancy compared with the general population remains reduced [7,8], with a standardized mortality ratio of 2.7:1 reported for Addison’s disease and from 1.73:1 to 2.17:1 for hypopituitarism [9-13]. The causes behind the increased mortality in AI are not fully known, but current strategies for managing AI are increasingly recognized as inadequate [14], with an overall frequency of 6.3 adrenal crises per 100 patient-years [15]. In addition, a recent report has indicated that hypocortisolism during acute stress causes deaths among adult patients with pituitary insufficiency [16]. Conventional glucocorticoid replacement therapy has also been associated with reduced bone mineral density [17], depending on the dose [18], as well as with unfavourable metabolic effects [19] and an increased risk of cardiovascular disease [20]. Moreover, suboptimal glucocorticoid replacement and the associated morbidities may contribute to a poor quality of life, characterized by negative alterations in physical activity and social, work and family life [5,21-23].

A novel once-daily modified-release preparation of hydrocortisone (Plenadren^®^, ViroPharma SPRL, Brussels, Belgium) has recently been approved for the treatment of AI in adults. This formulation has a dual-release mechanism, achieved by means of an immediate-release fraction of hydrocortisone in the outer layer of the tablet and an extended-release fraction in the core [24]. Administration of this modified-release preparation generates a plasma cortisol profile that mimics the natural diurnal pattern of circulating cortisol, except for the early morning increase in cortisol that occurs during sleep [25]. Results of a prospective randomized trial have shown that once-daily treatment with modified-release hydrocortisone provides a sustained serum cortisol concentration for the first 4 hours after the morning dose, followed by a steady reduction, which more closely approximates to the physiological plasma profile than is achieved with conventional thrice-daily dosing [6]. In addition, 85% of all patients expressed a ‘large’ or ‘very large’ preference for once-daily compared with thrice-daily hydrocortisone treatment, and over 90% of patients with diabetes mellitus expressed a preference for once-daily treatment [6]. It is therefore reasonable to expect that, in practice, once-daily dosing would impact positively on compliance compared with the twice- or thrice-daily regimens of conventional therapy.

The European Adrenal Insufficiency Registry **(**EU-AIR) is a unique European, multicentre, multinational, post-authorization, observational study established to collect long-term data on the safety of modified-release hydrocortisone and other glucocorticoid replacement therapies used in normal clinical practice (ClinicalTrials.gov identifier: NCT01661387). The study was designed to meet a regulatory requirement of the European Medicines Agency (EMA) for long-term safety data. The EU-AIR protocol also provides an opportunity to analyse data beyond those required by the EMA, including comparison of different replacement regimens in different subgroups of patients (e.g. those with diabetes mellitus and/or hypertension). The present paper describes the protocol for EU-AIR, the current status of the study and preliminary baseline data.

## Methods

### Study objectives

The main objective of EU-AIR is to monitor the safety of long-term treatment with once-daily modified-release hydrocortisone and other glucocorticoid replacement therapies in patients with AI. The registry focuses on intercurrent illness, adrenal crisis and serious adverse events (SAEs). Data relating to adverse drug reactions (ADRs) and mortality are also recorded. Safety data for specific subgroups, including those with hypertension and/or diabetes, hepatic or renal impairment or disorders of gastrointestinal emptying or motility, as well as pregnant or lactating women and the elderly will be examined.

### Study design

**Figure 1:**
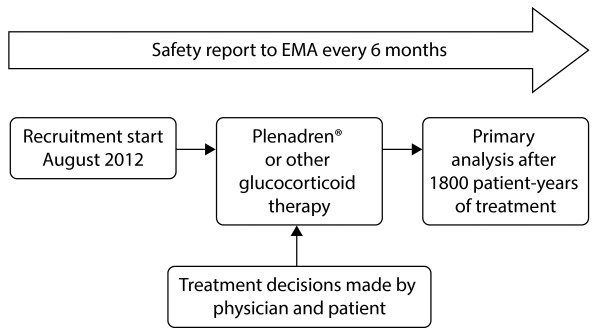
**Flow diagram of the EU-AIR study design.** EMA, European Medicines Agency.

EU-AIR is an observational study in patients with primary AI, secondary AI or congential adrenal hyperplasia who are undergoing long-term treatment with modified-release hydrocortisone or other glucocorticoid replacement therapies. Figure 1 provides an overview of the study. Data are collected from endocrinology centres in Germany, the Netherlands, Sweden and the UK. All enrolled patients will be followed during the course of routine clinical practice for the active duration of the registry. All medical care decisions, including those relating to treatment choice, are entirely at the discretion of the patient and registry physician.

The main endpoints of the study are the incidence rates of intercurrent illness events, adrenal crisis events and SAEs, as well as the duration of SAEs and glucocorticoid dose changes related to SAEs.

Patient data are collected by means of an electronic case report form (eCRF) at enrolment, and thereafter at routine clinic visits (every 6–12 months). More frequent visits to the registry physician can be made in cases of complications (e.g. adrenal crisis) or when changes in glucocorticoid replacement therapy are required.

The study is open ended; a decision to terminate it would be based on mutual agreement between the study sponsor and the Committee for Medicinal Products for Human Use.

### Study population

All patients with a diagnosis of AI at participating clinics and who are receiving modified-release hydrocortisone or other glucocorticoid replacement therapies are eligible for inclusion in the study. Any patients participating in an interventional clinical study are excluded and must wait for 3 months after completion of the interventional study before enrolling in EU-AIR.

All enrolled patients or their parent(s)/legal guardian(s) are required to provide written informed consent/assent. The study protocol has been approved by each country in which patients are enrolled, and then cascaded down to the individual centres. Ethical Committee approval in the Netherlands is currently pending. In Germany, only adult patients with AI are eligible for inclusion in the study.

### Treatments

All decisions regarding treatment and patient care are made by the physician and/or patient. No guidance is given on these issues in the study protocol.

### Patient withdrawal

A patient may discontinue their involvement in the registry either at their own request or if it is the opinion of the registry physician that it is not in the patient’s best interest to continue. Reasons for discontinuation are recorded on the eCRF. Patients who discontinue modified-release hydrocortisone are encouraged to continue in the registry unless consent has been withdrawn.

### Data collection

The data recorded in the eCRF at baseline and during subsequent routine clinic visits are listed in Table 1. Laboratory assessments conducted at baseline and at routine clinic visits are listed in Table 2.

**Table 1:** Data collection at baseline and at routine clinic visits

	**Baseline**	**Routine clinic visits**^**a**^
Informed consent	√	×
Demographic information	√	×
AI diagnosis (aetiology and year of diagnosis)	√	×
Relevant medical history	√	×
Relevant concomitant disease^b^	√	√
Glucocorticoid replacement therapy^c^	√	√
Concomitant medication	√	√
Physical examination^d^	√	√
Vital signs^e^	√	√
Pregnancy status^f^	√	√
Laboratory assessments^g^	√	√
DEXA	√^h^	√^i^

^a^Approximately every 6 months or more frequently in cases of complications (e.g. adrenal crisis) or when changes to treatment are required.

^b^Diabetes, hypertension, other hormone deficiencies, renal/hepatic impairment and disorders/diseases of gastrointestinal emptying/motility; year of diagnosis to be recorded.

^c^Glucocorticoid replacement therapy to be specified (generic name), plus dosing regimen and start/stop dates. Patients are defined as naive or established on their current therapy on the basis of the threshold of < 90 days and ≥ 90 days of treatment exposure, respectively.

^d^Height (baseline only), body weight and waist circumference.

^e^Blood pressure (systolic and diastolic) and heart rate.

^f^Recorded at baseline, if available; as applicable post-baseline.

^g^Sodium, potassium and glycated haemoglobin are recorded in the eCRF at enrolment, if available; see Table 2 for other laboratory parameters to be recorded during the study, as available.

^h^Recorded in the eCRF at baseline in patients identified as at high risk in centres routinely performing DEXA.

^i^As performed during routine clinic visits (every 2–3 years in patients identified as at high risk).

AI, adrenal insufficiency; DEXA, dual-energy X-ray absorptiometry; eCRF, electronic case report form.

**Table 2:** Laboratory assessments at baseline and at routine clinic visits (as available)

	**Baseline**	**Routine clinic visits**^**a**^
Fasting plasma glucose	√	√
Fasting insulin	√	√
Glycated haemoglobin A1c	√	√
Serum triglycerides	√	√
Cholesterol	√	√
High-density lipoprotein	√	√
Low-density lipoprotein	√	√
Apolipoprotein B/apolipoprotein A1	√	√
Serum sodium	√	√
Serum potassium	√	√
Serum renin	√	√
Serum osteocalcin	√	√
Serum procollagen I *N*-terminal propeptide	√	√

^a^Approximately every 6 months, or more frequently in cases of complications (e.g. adrenal crisis) or when changes to treatment are required.

Where dual-energy X-ray absorptiometry measurements are made in routine clinical practice, or in patients deemed at high risk of osteoporosis, these data will be recorded. Patients are also monitored for intercurrent illness, adrenal crisis, SAEs (any serious event not necessarily associated with the study medication) and ADRs (considered to be associated with the study medication). Investigators are responsible for determining whether an intercurrent illness or adrenal crisis has occurred, and for determining the duration of the event and any associated change in glucocorticoid dose. Intercurrent illness is defined as any temporal illness for which a transient significant increase in glucocorticoid replacement dose (e.g. doubling the dose) is needed. Non-disease conditions, including mental or physical stress, such as vigorous exercise, which might be covered by an extra 5 mg dose of hydrocortisone, for example, are excluded. The definition of an adrenal crisis was agreed with the EMA. Specifically, an adrenal crisis was defined as an acute impairment of general health with the need for parenteral hydrocortisone and saline infusion. For any ADR or SAE, the registry physician is required to evaluate and report the onset date, outcome, end date, severity (mild, moderate, severe or life-threatening/disabling), relationship to glucocorticoid replacement therapy, actions taken, seriousness and any change in glucocorticoid dose (and its duration). Deaths are recorded as outcomes rather than ADRs.

If a pregnant woman enters the study, or a female patient becomes pregnant during the study, the patient is requested to inform the registry physician immediately.

Patients are provided with paper-based diaries at enrolment, in which they are asked to record any health issues that occur between clinic visits, as well as illness-related glucocorticoid dose changes and reasons for such dose changes. Patients are also asked to use these diaries to record details of any medication they receive in addition to recording their AI treatment.

### Sample size

The size of the registry has been calculated to evaluate the non-inferiority of modified-release hydrocortisone relative to conventional hydrocortisone replacement therapy. Assuming a rate of four events of intercurrent illness per patient-year and specifying a non-inferiority limit of 0.2 events per patient-year (i.e. 5% of the incidence rate), an exposure of 1800 patient-years in each treatment group (i.e. 3600 patient-years in total) will yield 80% power to demonstrate non-inferiority of modified-release hydrocortisone relative to conventional glucocorticoid replacement therapy. It is anticipated that this level of exposure will have been achieved approximately 3 years after the start of the registry. The validity of these design assumptions will be re-assessed after 900 patient-years of exposure in each treatment group. Power calculations employing the observed incidence of events and dropout rates will be performed. Timing of the principal analysis may be revised in the light of those calculations.

### Statistical analysis

Results will be analysed by treatment group (i.e. modified-release hydrocortisone versus other glucocorticoid replacement therapies) and in total. SAEs and ADRs will be summarized by individual event and system organ class.

A test of the null hypothesis that modified-release hydrocortisone is less safe than other glucocorticoid replacement therapies for the incidence of intercurrent illness in patients with AI will be performed when the registry contains data for at least 1800 patient-years of observational time in each treatment group. This test will examine the upper limit of a 95% confidence interval (CI) for the incidence rate difference in intercurrent illness by comparison with the non-inferiority limit (i.e. 5% of the incidence rate). Non-inferiority will be demonstrated if the upper limit of this CI is less than the non-inferiority limit. This methodology will also be employed to evaluate the non-inferiority of modified-release hydrocortisone with respect to the incidence of adrenal crisis. For any test of non-inferiority that is deemed significant, a subsequent test of superiority will be performed.

### Reporting data

Reports from the registry, incorporating data up to and including the most recent clinic visit for each patient, will be provided to the EMA on a regular basis.

The registry physician, study coordinator, other designated study personnel or study monitor is required to report any SAE to ViroPharma Drug Safety within 24 hours of becoming aware of the event. Registry physicians are responsible for reporting SAEs to their respective independent reviewing authority, institutional review board or independent ethics committee according to local reporting requirements. Cases of accidental exposure, overdose, other medication errors and pregnancy/lactation exposure are also required to be reported to ViroPharma Drug Safety.

## Results

**Figure 2:**
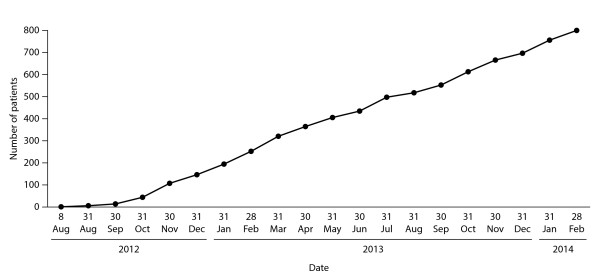
Cumulative numbers of patients enrolled in EU-AIR since August 2012.

The cumulative number of patients in EU-AIR since August 2012 is shown in Figure 2. Baseline demographic data available for patients enrolled by March 2014 (n = 801) are shown in Table 3 (owing to the lag between enrolment and data entry at study sites, some demographic data are currently incomplete). Of the 801 patients, 301 had primary AI, 454 secondary AI and 46 CAH. Mean age (±SD) at enrolment was similar for patients with primary and secondary AI (51.6 ± 15.9 years and 54.1 ± 16.6 years, respectively) but lower for patients with CAH (37.3 ± 13.0 years).

**Table 3:** Demographic details of patients enrolled in EU-AIR as of March 2014

	**All AI**	**Primary AI**	**Secondary AI**	**CAH**
Number	801	301	454	46
Age (years)	52.2 (12–87)	51.6 (17–86)	54.1 (12–87)	37.3 (12–71)
Gender				
Male	353	99	236	18
Female	448	202	218	28
BMI (kg/m^2^)				
Male	28.5 (18.7–48.9)	26.5 (18.7–48.9)	29.4 (18.9–43.2)	26.7 (19.6–39.8)
Female	27.7 (17.9–56.4)	26.1 (17.9–43.0)	29.0 (17.9–55.6)	29.4 (18.4–56.4)

Values are means, with the range given in parentheses.

AI, adrenal insufficiency; BMI, body mass index; CAH, congenital adrenal hyperplasia.

At enrolment, 663 patients were receiving conventional hydrocortisone replacement, 62 were receiving prednisolone and 16 dexamethasone. Information for the remaining 60 patients is currently not available in the database.

## Discussion and conclusions

Randomized controlled trials (RCTs) are generally considered the gold standard for generating safety and efficacy data in the assessment of pharmaceutical utility. However, restrictive entry criteria and stringent protocols can be barriers to generalizing RCT findings to clinical practice. Although observational studies lack the robustness of RCTs and may include uncontrolled confounding variables [26], they can nevertheless yield important data to complement the results from RCTs (e.g. in the study of rare outcomes, risk factors and side effects) and provide valuable information on long-term outcomes in routine clinical practice [27]. As EU-AIR plans to enrol many more patients than would be possible in an RCT, some weaknesses of the observational study design may be offset by the increase in information expected from a large study population. In addition, the constraints of rigid exclusion and inclusion criteria applied to RCTs may limit the ability to extrapolate the results to a wider population. In contrast, EU-AIR has no obstacles to enrolment, other than the requirement that participants are receiving glucocorticoid replacement therapy. This feature allows, dependent on local approval, the participation of pregnant women, children and other patients with AI who would not qualify for inclusion in an RCT.

EU-AIR will provide extensive outcomes data over a longer time period than the randomized crossover trial that preceded it [6]. The extent to which RCT data are representative of outcomes obtained in routine clinical practice may be particularly pertinent in the case of AI, given the complexity of the conventional dosing regimen, patient self-adjustment of the dose, compliance issues with prescribed treatment regimens and the difficulties associated with optimizing maintenance dosing [28]. It is expected that once-daily dosing with modified-release hydrocortisone will be associated with improved compliance when compared with conventional hydrocortisone regimens that require more frequent dosing. It may also be possible to evaluate the relationship between treatment compliance and clinical outcomes, which would be difficult to achieve in a conventional RCT design.

Large-scale observational studies have demonstrated their value in enhancing understanding of other rare conditions and treatments. The pharmacoepidemiological survey known as the Pfizer International Metabolic Database (KIMS) has been established for nearly 20 years, during which time it has proven itself to be a valuable tool for monitoring the use, efficacy and safety of growth hormone (GH) treatment in adults with GH deficiency [29]. For example, it was shown that 2 years of GH replacement therapy reduced the risk of cardiovascular events by 50%, and that male sex and high total cholesterol were potential predictors of this response [30]. Similarly, the Fabry Outcome Survey (FOS), established in 2001 as an industry-sponsored, physician-directed registry of patients with Fabry disease (FD), a rare lysosomal storage disorder, has made an important contribution to the understanding of both the disease and the patients’ real-world experience of enzyme replacement therapy [31]. In other rare diseases, registries have proven valuable for revealing shortcomings of current treatment (e.g. in patients with adrenocortical cancer [32] or primary hyperaldosteronism [33]).

In addition to the comparison of modified-release hydrocortisone with traditional glucocorticoid replacement (hydrocortisone, cortisone acetate, prednisolone, dexamethasone), the design of EU-AIR will allow assessment of the range of treatments employed for AI of differing aetiologies and in specific patient subpopulations (e.g. those with hypertension, diabetes and/or other hormone deficiencies). The detail recorded in the patient diary is such that data may also be classified by prescribed drug, dose and frequency of administration. These aspects of the comparative design of this registry allow for predefined statistical analysis, thus ensuring scientific robustness in the observational setting. Within EU-AIR it is also possible to gather health economic and resource utilization information in addition to the required safety data. Together, these data will generate valuable insights into the benefits of different cortisol replacement therapies in routine clinical practice.

Although it does not replicate a complete 24-hour physiological cortisol profile (notably, it does not accurately mimic the early morning cortisol peak [25]), modified-release hydrocortisone represents a significant step towards achieving a diurnal plasma profile more similar to the normal circadian pattern than is possible with standard glucocorticoid replacement therapy. Thus, modified-release hydrocortisone is expected to resolve a number of the shortcomings associated with conventional cortisol replacement therapy, which has remained essentially unchallenged for over 50 years, despite being associated with considerable unmet needs. EU-AIR should provide an appropriate setting to assess long-term morbidity and mortality trends in patients receiving modified-release and other glucocorticoid therapies, including parameters such as compliance and quality of life that are difficult to evaluate effectively in the setting of a controlled trial.

In summary, EU-AIR is anticipated to become a valuable source of information about current approaches to treating AI as well as the long-term safety and tolerability of both modified-release and conventional glucocorticoid replacement therapy. In addition to addressing regulatory requirements, this registry will be an enduring data source for improving the management of AI, as has been the case with similar databases established for other rare conditions.

## Competing interests

PMJZ and MQ are members of the International Plenadren® Advisory Board for ViroPharma and the EU-AIR Scientific Steering Committee, and have received fees for consultancy from ViroPharma. RDM is a member of the UK Advisory Board and EU-AIR Scientific Steering Committee and has received fees for consultancy from ViroPharma. BE is a member of the Swedish Advisory Board and EU-AIR Scientific Steering Committee and has received fees for consultancy from ViroPharma. CM and DF are employees of ViroPharma.

## Authors’ contributions

All authors contributed to the development of the manuscript, and all approved the final version for publication. BE, RDM, MQ and PMJZ are Principal Investigators for the countries involved in EU-AIR and were instrumental in the design of the Registry. DF was responsible for overall data analysis and CM for study design and coordination.

## Pre-publication history

The pre-publication history for this paper can be accessed here:

http://www.biomedcentral.com/1472-6823/14/40/prepub

## UK

Dr Murray, Leeds, Prof Pearce, Newcastle, Prof Grossman, Oxford, Prof Stewart, Birmingham, Prof Toogood. Birmingham, Prof Bouloux, London and Prof Drake, London, Prof Trainer, Manchester.

## Sweden

Dr Ekman, Linköping and Dr Nilsson, Gothenburg.

## Germany

Dr Quinkler, Berlin, Prof Stalla, Munich, Prof Beuschlein, Munich, Prof Schoefl, Erlangen, Dr Hahner, Wuerzburg and Prof Badenhoop, Frankfurt.

## The Netherlands

Dr Zelissen, Utrecht, Prof Pereira, Leiden, Dr van Beek, Groningen and Dr Feelders, Rotterdam.

The authors acknowledge the editorial support of Dr David Campbell, Oxford PharmaGenesis™ Ltd.
